# Short-term choroidal vascular changes after aflibercept therapy for neovascular age-related macular degeneration

**DOI:** 10.1007/s00417-020-04957-5

**Published:** 2020-10-13

**Authors:** Marco Pellegrini, Federico Bernabei, Andrea Mercanti, Stefano Sebastiani, Enrico Peiretti, Claudio Iovino, Giamberto Casini, Pasquale Loiudice, Vincenzo Scorcia, Giuseppe Giannaccare

**Affiliations:** 1 Ophthalmology Unit, S.Orsola-Malpighi University Hospital, University of Bologna, Via Palagi 9, 40138 Bologna, Italy; 2 Ophthalmology Unit, Head and Neck Department of Ophthalmology, Infermi Hospital, Rimini, Italy; 3 Department of Surgical Science, Eye Clinic, University of Cagliari, Cagliari, Italy; 4 Eye Clinic, Multidisciplinary Department of Medical, Surgical and Dental Sciences, Università degli Studi della Campania ‘Luigi Vanvitelli’, Naples, Italy; 5 Ophthalmology Unit, Department of Surgical, Medical, Molecular and Critical Area Pathology, University of Pisa, Pisa, Italy; 6 Department of Ophthalmology, University of “Magna Graecia”, Catanzaro, Italy

**Keywords:** Age-related macular degeneration, Anti-VEGF, Choroidal vascularity index, Optical coherence tomography

## Abstract

**Introduction:**

The purpose of this study was to evaluate choroidal vascular changes in patients with neovascular age-related macular degeneration (nAMD) treated with aflibercept injection over a 3-month period.

**Methods:**

Enhanced depth imaging optical coherence tomography scans of 60 eyes with treatment-naïve nAMD and 60 unaffected fellow eyes were retrospectively analyzed. Data was collected at baseline and after 3 monthly intravitreal injections of aflibercept. The ImageJ software was used to binarize OCT scans and measure total choroid area (TCA), luminal area (LA), and stromal area (SA). Choroidal vascularity index (CVI) was defined as the ratio of LA to TCA.

**Results:**

After treatment, subfoveal choroidal thickness (CT) in nAMD eyes significantly decreased from 210. 6 ± 61.6 to 194.6 ± 58.7 μm (*P* < 0.001), TCA from 1.620 ± 0.502 to 1.500 ± 0.451 mm^2^ (*P* < 0.001), LA from 1.075 ± 0.335 to 0.985 ± 0.307 mm^2^ (*P* < 0.001), SA from 0.545 ± 0.176 to 0.516 ± 0.153 mm^2^ (*P* = 0.005), and CVI from 66.36 ± 2.89 to 65.46 ± 2.87% (*P* = 0.009). The decrease of CVI after treatment was significantly correlated with baseline CVI (Rs = 0.466, *P* < 0.001), but not with the change in BCVA and presence of dry macula after treatment (always *P* > 0.05).

**Conclusion:**

Choroidal thickness and vascularity significantly decreased after treatment with aflibercept in nAMD eyes. Besides the pharmacologic effect on the neovascular lesion, aflibercept may induce vascular changes also on the underlying choroid.

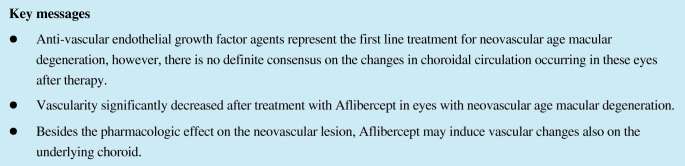



## Introduction

Age-related macular degeneration (AMD) is a progressive degenerative disease of the macula that represents the leading cause of legal blindness among elderly patients in developed countries [1]. Currently, intravitreal injection of anti-vascular endothelial growth factor (VEGF) agents is the first-line treatment for neovascular AMD (nAMD) in order to suppress exudation induced by choroidal neovascularization (CNV) [2–4]. Aflibercept (Eylea; Regeneron, Tarrytown, NY, USA, and Bayer, Leverkusen, Germany) is a recombinant soluble fusion protein consisting of the binding portions of VEGF receptors 1 and 2 fused to the constant region of an immunoglobulin G-1. This agent acts as a decoy receptor for all isoforms of VEGF-A, VEGF-B, and placental growth factor [5, 6]. Similar efficacy and safety outcomes as monthly ranibizumab (Lucentis; Genentech, Inc., South San Francisco, USA) were determined with intravitreal aflibercept dosed either monthly or every 2 months, following three initial loading doses [7].

Besides the effect on CNV, anti-VEGF agents may exert a pharmacologic action also on the choroid. However, to date, there is no definite consensus on the changes in choroidal circulation occurring in eyes with nAMD after anti-VEGF therapy. In fact, on one hand, previous reports demonstrated that subfoveal choroidal thickness (CT) decreases significantly after intravitreal injections of ranibizumab [8, 9] and aflibercept [10–12]. On the other hand, other studies did not find any significant changes in CT after intravitreal injections of bevacizumab (Avastin; Genentech, Inc., South San Francisco, USA) [13] and ranibizumab [10, 14, 15]. These inconsistencies may be due to some intrinsic limitations of CT measurement, which is affected by numerous factors including age, sex, systolic blood pressure, intraocular pressure, axial length, and refractive error [16, 17].

Recently, the introduction of image binarization techniques applied to enhanced depth imaging optical coherence tomography (OCT) and swept source OCT have enabled in vivo quantitative analysis of choroidal vascularity. Specifically, choroidal vascularity index (CVI), the ratio of the luminal to the cross-sectional choroidal area, seems to be a more reliable tool to assess choroid vascular status compared with CT, since it shows lower variability and is influenced by fewer physiologic factors [18–21].

The purpose of this study was to investigate the choroidal vascular changes with image binarization of OCT scans in eyes with nAMD treated with aflibercept injection over a 3-month period.

## Materials and methods

This retrospective study included patients with treatment-naïve unilateral nAMD seen at the Department of Ophthalmology of the University of “Magna Graecia” of Catanzaro (Italy) between January 2017 and June 2019. The study was performed in accordance with the principles of the Declaration of Helsinki and was approved by the local Ethics Committee.

All patients underwent a comprehensive ophthalmic evaluation including best-corrected visual acuity (BCVA), slit-lamp biomicroscopy, fundoscopy, fluorescein angiography, indocyanine green angiography (ICGA), and spectral domain OCT (Heidelberg Retina Angiography, Spectralis; Heidelberg Engineering, Heidelberg, Germany). Diagnosis of nAMD was based on fundoscopy, fluorescein angiography, and ICGA. Patients with a diagnosis of polypoidal choroidal vasculopathy (PCV), based on the presence of protruded orange-red elevated lesions on fundoscopy and/or characteristic polypoidal lesions seen in ICGA [22], were excluded from the study. Other exclusion criteria were history of any other retinal diseases (e.g., central serous chorioretinopathy, diabetic retinopathy, and retinal dystrophy), history of photodynamic therapy and pars plana vitrectomy, a spherical equivalent of –6 diopters or less, and/or chorioretinal atrophic changes secondary to pathologic myopia, presence or history of nAMD in the fellow eye, poorly visible choroidal–scleral junction on OCT, and hemodynamically significant carotid stenosis. The untreated fellow eyes were used as controls.

All patients were treated with three consecutive monthly 2.0-mg intravitreal injections of aflibercept. After the last injection, ophthalmic evaluation including BCVA testing, slit-lamp biomicroscopy, fundoscopy, and OCT imaging was repeated. All OCT scans were obtained almost at the same time of the morning (9 am), during the daily clinical activity. A macula was judged dry in case of absence of subretinal and intraretinal fluid on OCT after treatment. The subfoveal CT was measured manually using the caliper function tool of the image analysis software.

**Fig. 1:**
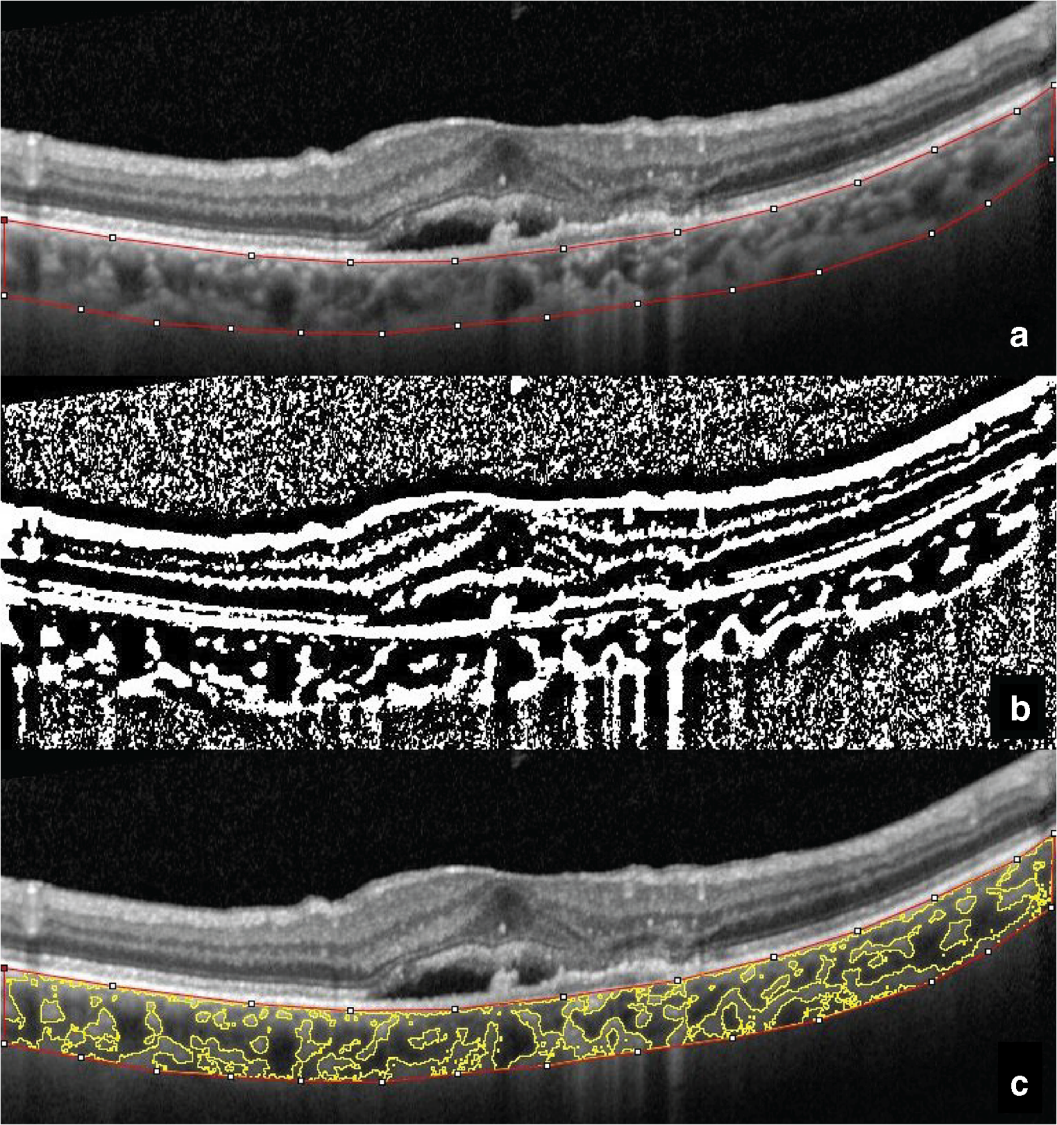
Choroidal vascularity index calculation with binarization of spectral-domain OCT images. **a** Choroidal boundaries are traced to identify the total choroidal area (red lines). **b** The image is binarized with Niblack’s auto-local threshold. **c** The color threshold tool is used to select the dark pixels, representing the luminal area (yellow lines). The choroidal vascularity index is computed dividing luminal area by total choroidal area

To measure CVI, the OCT scans passing through the fovea were selected and analyzed with the software ImageJ 1.51s (National Institutes of Health, Bethesda, MD, USA) by a masked examiner (M.P.) using a previously described protocol [23]. In brief, total choroidal area (TCA) was selected manually using the polygon tool. The entire length of the OCT scan was used in the segmentation of TCA (Fig. 1a). The upper boundary of the selection was traced along the choroid–retinal pigment epithelium junction and the lower boundary along the choroidal–scleral junction. After conversion to 8-bit images, Niblack’s auto local thresholding was applied to binarize the image (Fig. 1b). The color threshold was then applied, and the luminal area (LA), represented by the dark pixels within the TCA, was selected and measured. The LA was subtracted from TCA to obtain the stromal area (SA). Finally, CVI was calculated as the ratio of LA to the TCA (Fig. 1c).

Statistical analysis was conducted with SPSS statistical software (SPSS Inc, Chicago, Illinois, USA). The BCVA was converted to the logarithm of the minimum angle of resolution (logMAR) units before the analyses. The normality of data was assessed with The Shapiro-Wilk’s test. Continuous variables in the nAMD and fellow eyes were compared using a paired sample *t* test in case of normal distribution and nonparametric Wilcoxon test in case of non-normal distribution. The same tests were used to compare variables before and after treatment in both eyes. The correlations between baseline choroidal parameters in nAMD eyes and sex, age, intraocular pressure (IOP), and BCVA, as well as the associations between the change of CVI after treatment and the baseline CVI, change in BCVA, and presence of dry macula after treatment, were assessed by use of the Spearman’s rank correlation analysis. A *P* value < 0.05 was considered statistically significant.

## Results

Sixty patients with treatment-naïve nAMD were included in this study. The baseline demographic and clinical characteristics of patients are reported in Table 1.

**Table 1:** Baseline demographic and clinical characteristics of patients included in the study

Characteristic	nAMD patients
Age (years)	74.8 ± 7.8
Sex (m/f)	35:25
Intraocular pressure (mmHg)	15.8 ± 3.1
BCVA (logMAR)	0.56 ± 0.47

*nAMD* neovascular age-related macular degeneration, *BCVA* best-corrected visual acuity, *logMAR* logarithm of the minimum angle of resolution

All the patients received regularly three consecutive monthly intravitreal injections of aflibercept 2.0 mg. After treatment, the mean BCVA significantly improved from 0.55 ± 0.46 to 0.46 ± 0.44 logMAR (*P* = 0.001). In particular, the BCVA improved in 36 eyes (60.0% of the total), was unchanged in 18 eyes (30%), and worsened in 6 eyes (10.0%). Thirty-seven patients (61.7%) achieved a dry macula with resolution of subretinal and intraretinal fluid on OCT.

**Fig. 2:**
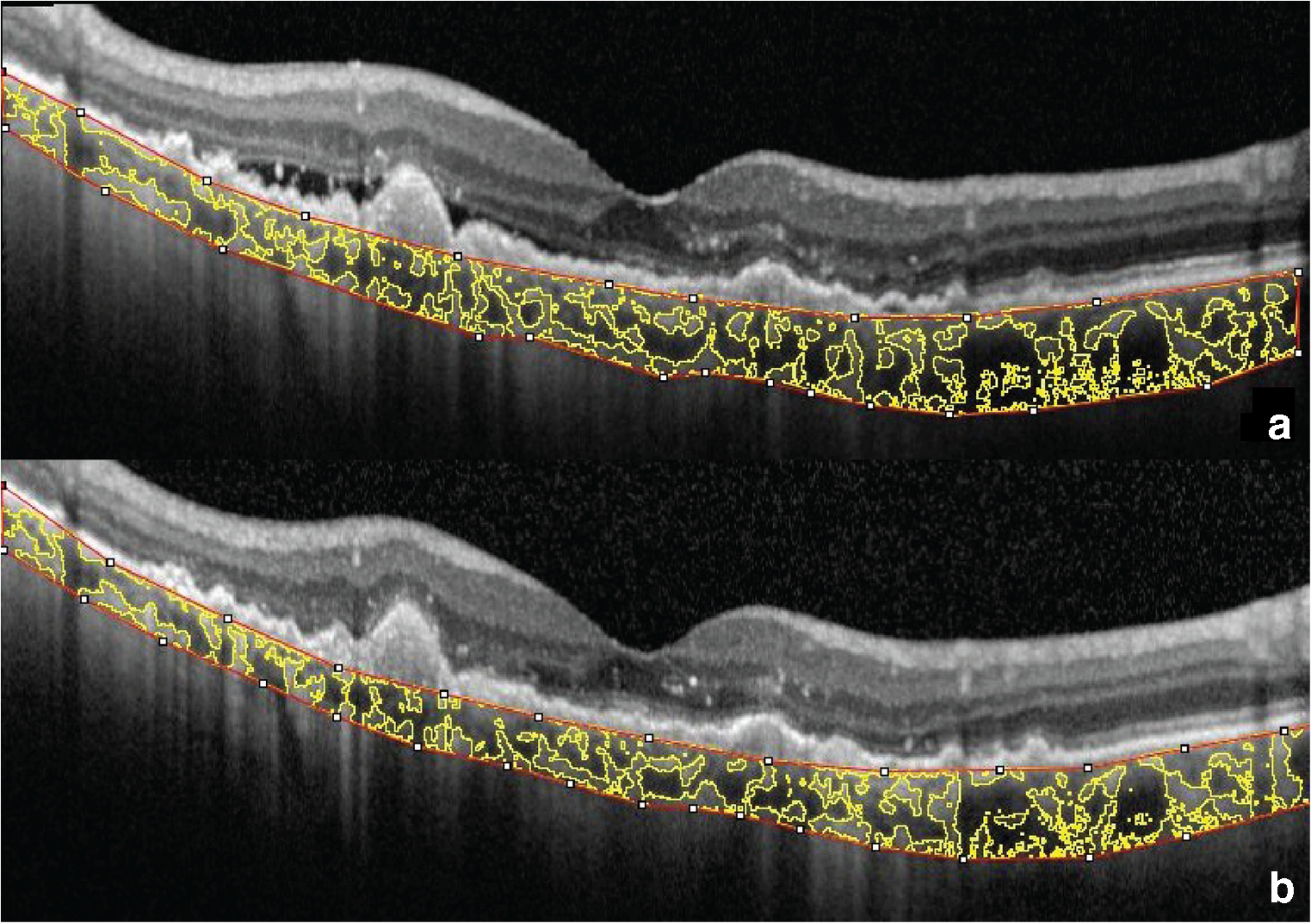
Enhanced depth imaging OCT with calculation of choroidal vascularity index in a representative eye with neovascular age-related macular degeneration at baseline (**a**) and after 3 monthly intravitreal injections of aflibercept (**b**)

Baseline choroidal parameters in eyes with nAMD and fellow eyes are reported in Table 2. Eyes with nAMD showed a higher subfoveal CT, TCA, and SA compared with fellow eyes (always *P* < 0.05). Conversely, LA and CVI did not significantly differ between nAMD and fellow eyes. After treatment, subfoveal CT in nAMD eyes significantly decreased from 210.6 ± 61.6 to 194.6 ± 58.7 μm (*P* < 0.001), TCA from 1.620 ± 0.502 to 1.500 ± 0.451 mm^2^ (*P* < 0.001), LA from 1.075 ± 0.335 to 0.985 ± 0.307 mm^2^ (*P* < 0.001), SA from 0.545 ± 0.176 to 0.516 ± 0.153 mm^2^ (*P* = 0.005), and CVI from 66.36 ± 2.89 to 65.46 ± 2.87% (*P* = 0.009). A representative case is shown in Fig. 2. Conversely, none of the choroidal parameters significantly changed in fellow eyes after treatment (always *P* > 0.05).

**Table 2:** Choroidal parameters in eyes with nAMD and fellow eyes

Characteristic	nAMD eyes	Fellow eyes		P
Baseline				
Subfoveal CT (μm)	210.6 ± 61.6	192.5 ± 62.2		0.005
TCA (mm^2^)	1.620 ± 0.502	1.490 ± 0.523		0.029
LA (mm^2^)	1.075 ± 0.335	1.003 ± 0.362		0.060
SA (mm^2^)	0.545 ± 0.176	0.487 ± 0.171		0.010
CVI (%)	66.36 ± 2.89	67.14 ± 3.11		0.088
After treatment				
Subfoveal CT (μm)	194.6 ± 58.7	193.7 ± 64.9		0.005
TCA (mm^2^)	1.500 ± 0.451	1.451 ± 0.497		0.029
LA (mm^2^)	0.985 ± 0.307	0.981 ± 0.346		0.060
SA (mm^2^)	0.516 ± 0.153	0.471 ± 0.157		0.010
CVI (%)	65.46 ± 2.87	67.37 ± 2.77		0.088

*nAMD* neovascular age-related macular degeneration, *CT* choroidal thickness, *TCA* total choroidal area, *LA* luminal area, *SA* stromal area, *CVI* choroidal vascularity index

**Fig. 3:**
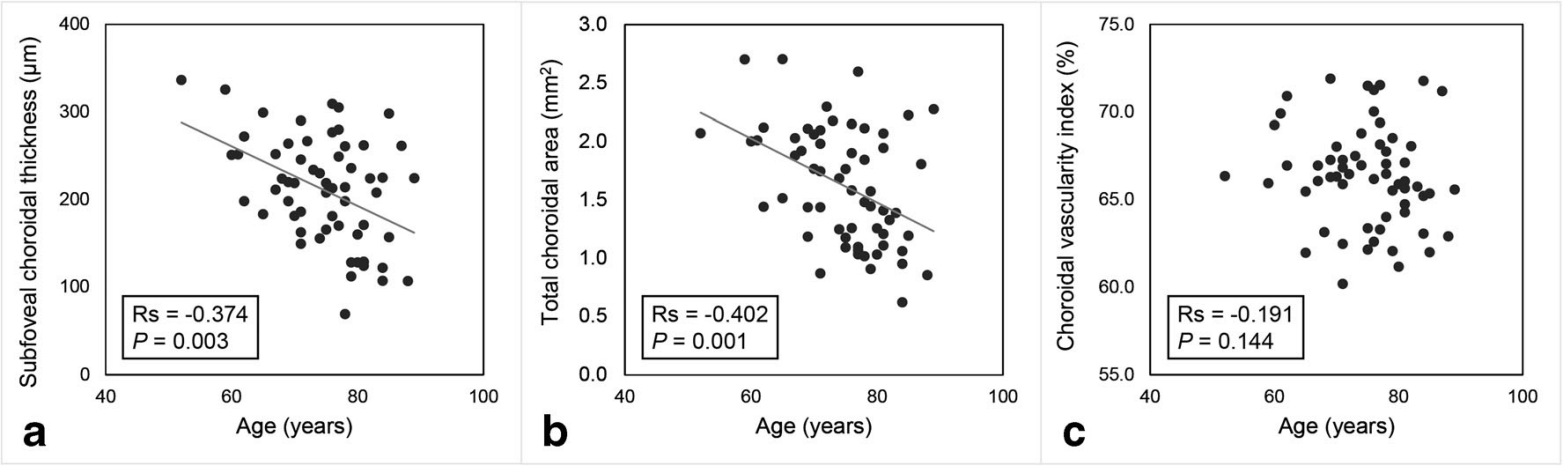
Scatterplots showing the relationship between age and subfoveal choroidal thickness (**a**), total choroidal area (**b**), and choroidal vascularity index (**c**) in eyes with neovascular age-related macular degeneration at baseline. Age was significantly correlated with subfoveal choroidal thickness and total choroidal area but not with choroidal vascularity index

**Fig. 4:**
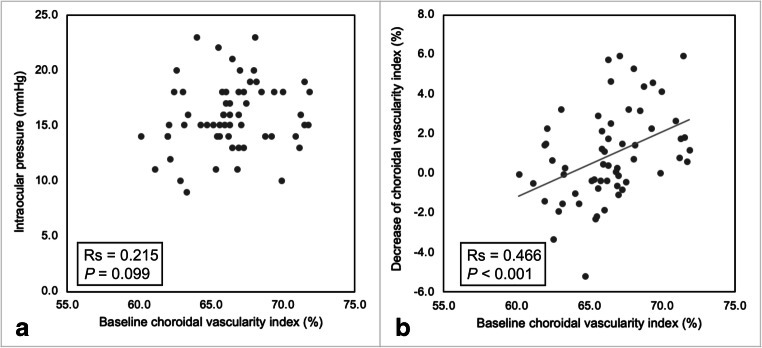
Scatterplots showing the relationship between choroidal vascularity index at baseline and intraocular pressure (**a**) and decrease of choroidal vascularity index after 3 monthly intravitreal injections of aflibercept (**b**) in eyes with neovascular age-related macular degeneration

In nAMD eyes, the age of the patients was negatively correlated with baseline subfoveal CT (Rs = − 0.374, *P* = 0.003), TCA (Rs = − 0.402, *P* = 0.001), LA (Rs = − 0.423, *P* = 0.001), and SA (Rs = − 0.346, *P* = 0.007), but not with CVI (*P* = 0.144) (Fig. 3). Similarly, in fellow eyes, the age was negatively correlated with baseline subfoveal CT (Rs = − 0.414, *P* = 0.001), TCA (Rs = − 0.343, *P* = 0.007), LA (Rs = − 0.348, *P* = 0.006), and SA (Rs = − 0.341, *P* = 0.008), but not with CVI (*P* = 0.525). No significant correlations between any baseline choroidal parameter and sex, IOP, and BCVA were found (always *P* > 0.05; Fig. 4a). The decrease of CVI after treatment was significantly correlated with baseline CVI (Rs = 0.466, *P* < 0.001) (Fig. 4b), but not with the change in BCVA and presence of dry macula after treatment (always *P* > 0.05). Similarly, the presence of dry macula did not correlate with the change of the other choroidal parameters (always *P* > 0.05).

## Discussion

In the present study, we evaluated the changes within sub-components of the choroid in eyes with treatment-naïve nAMD at baseline and after intravitreal aflibercept injections. As previously described, eyes with nAMD demonstrated a thicker choroid compared with fellow eyes before anti-VEGF treatment [24, 25]. Conversely, baseline CVI did not differ significantly between affected and control eyes, and the thicker choroid in nAMD eyes was associated with an increase of both the luminal and stromal components. This result was consistent with those of Koh et al, which also noted decreased choroidal vascularity in eyes with AMD as well as in normal fellow eyes of AMD patients in comparison with age-matched healthy subjects [24]. This result was interpreted as a sign of subclinical choroidal ischemia, which may precede the typical manifestations of AMD such as CNV. Another recent study described an increase of choroidal vascularity in eyes with AMD before development or reactivation of CNV [26]. Therefore, serial CVI measurement might be a useful tool to predict the development or reactivation of CNV in patients with AMD.

After three monthly intravitreal injections of aflibercept, 90% of the eyes included in this study had improved or stable BCVA, with a 61.7% rate of dry macula. Furthermore, the mean subfoveal CT significantly decreased in treated eyes. In addition, the binarization analyses of the choroid showed a significant reduction of TCA, LA, SA, and CVI. Since CVI represents the proportion of LA and TCA, its decrease implies a higher reduction of the vascular choroidal component compared with the stromal one. Conversely, none of the choroidal parameters significantly changed in the untreated fellow eyes.

Several previous studies have documented choroidal thinning after anti-VEGF therapy with both ranibizumab [8, 9] and aflibercept [10–12]. Koizumi et al demonstrated that most of the thinning occurs after the first 3-month injections and is followed by a plateau out to 12 months with bimonthly injections [12]. Another study reported that the initial choroidal thinning caused by anti-VEGF treatment was maintained in patients who required further anti-VEGF administration, while it was followed by a progressive re-thickening in those who did not undergo additional injections [27]. In addition, a decrease of subfoveal choroidal blood flow after intravitreal ranibizumab in eyes with nAMD was demonstrated by using laser Doppler flowmetry [28]. However, limited data are available on the changes of the CVI occurring after intravitreal aflibercept [29].

To our knowledge, only a previous study evaluated CVI in eyes with nAMD and PCV following anti-VEGF therapy and reported no significant changes from baseline to month 12 [29]. This finding was not consistent with the results of the present work. However, there were important differences in cohorts between the two studies: in contrast to Ting et al, we only included eyes treated with aflibercept, which is believed to induce greater effect on the choroid compared with the other anti-VEGF agents [10]. Furthermore, we decided to exclude patients with PCV in order to obtain a homogeneous population, since eyes with PCV may have distinct choroidal features leading to CNV development, such as hyperpermeability and dilation of outer choroidal vessels, as well as a different response to anti-VEGF therapy compared with typical nAMD [30]. In addition, the large pigment epithelial detachments seen in eyes with PVC have extensive shadowing effect on the underlying choroid, and this may affect the accuracy of OCT binarization and CVI calculation [29].

Although the choroidal pharmacokinetics of anti-VEGF agents has yet to be clarified, aflibercept may have a direct effect not only on the neovascular lesion, but also on the underlying choroid. VEGF-A has numerous pharmacologic actions, including stimulation of angiogenesis, increase in microvascular permeability, and dilation of vessels [31]. The suppression of VEGF is associated with reduction of choriocapillaris endothelial cell fenestrations [32] and may lead to choroidal thinning by reduction of choroidal vascular permeability and/or by vasoconstriction [12]. The decrease of CVI observed in this study seems to support this hypothesis. Another possibility is that the choroidal changes after anti-VEGF treatment may be secondary to the suppression of the CNV activity and leakage. Although the OCT scanning area has little effects on the CVI measurement [33], the vascular changes secondary to anti-VEGF treatment might affect the whole choroid, and not only the subfoveal region. Thus, in the present study, the choroidal vascularity was measured across the entire length of the OCT scan.

The subfoveal choroid in normal eyes naturally thins with aging by approximately 15 μm with every decade of life [34]. Conversely, CVI does not seem to have a significant relationship with age [19]. In agreement with these observations, we found that the age was negatively correlated with subfoveal CT and TCA, but not with CVI in both nAMD and fellow eyes. This suggests that aging is associated with a reduction of both the vascular and stromal choroidal components, which may be relatively independent from the presence of nAMD. Moreover, eyes with high baseline CVI showed greater CVI reduction after anti-VEGF treatment. A similar result was reported by Ting and colleagues, who speculated that differences in choroidal vascularity might reflect different pathogenic processes and choroidal remodeling in eyes with and without pachychoroid and choroidal hyperpermeability [29].

The clinical significance of the choroidal changes occurring in eyes with nAMD after anti-VEGF treatment remains unclear. Previous reports showed that the decrease in subfoveal CT was greater in eyes without persistent or recurrent retinal fluid [11] and was related to visual acuity improvement in PCV, but not in typical nAMD [12]. On the other hand, a certain level of VEGF is required to maintain the perfusion of the choriocapillaris, which is the major source of nutrients and oxygen for the outer retina [35]. A recent study reported a decrease in the choriocapillaris vascular density during long-term anti-VEGF therapy for nAMD [36]. Therefore, excessive choroidal thinning and decreased vascularity after anti-VEGF therapy may be an undesired effect leading to deterioration in the choriocapillaris-RPE complex and development of geographic atrophy [37, 38]. In the present study, we could not identify any association between choroidal changes and anatomical and visual outcomes. For this matter, the functional prognostic value of CVI needs further investigation in long-term prospective studies.

The main limitations of this study are related to the short follow-up period, which make it difficult to draw definitive conclusions. In addition, the retrospective design hampered the evaluation of potentially confounding variables such as axial length, blood pressure, and smoking status, all of which may affect choroidal parameters [19].

In conclusion, CVI decreased in eyes with nAMD treated with aflibercept. Intravitreal treatment with anti-VEGF may provide a pharmacologic effect not only on the CNV but also on the underlying choroid, reducing its thickness and vascularity. Although the clinical significance of these vascular changes *has still to be elucidated,* choroidal vascularity index may be a helpful tool in the monitoring of patients undergoing anti-VEGF treatment for nAMD. Further prospective studies are required to confirm the results of the current study and elucidate the long-term effect of aflibercept on the choroidal circulation.

## Data Availability

The data that support the findings of this study are available on request from the corresponding author.
